# Metabolic Checkpoints in CD8^+^ T Cells within the Tumor Microenvironment: A Comprehensive Review and Emerging Insights

**DOI:** 10.7150/ijbs.125206

**Published:** 2026-01-22

**Authors:** Jiaqi Ai, Yiheng Du, Qianqian Xue, Wenbei Peng, Qiong Zhou

**Affiliations:** Department of Respiratory and Critical Care Medicine, Union Hospital, Tongii Medical College, Huazhong University of Science and Technology, Wuhan, 430022, China.

**Keywords:** cancer, CD8^+^ T cell, metabolism, checkpoint, immunotherapy

## Abstract

In recent years, a growing number of evidence suggests that cancer is a metabolic disease. Metabolic reprogramming is a hallmark of the TME, where various nutrients, including glucose, lipids, and amino acids, play key roles in regulating tumor development by acting on both tumor cells and immune cells. T cells are the core mediators of anti-tumor immunity. Especially CD8^+^ T cells are considered the primary immune cells involved in the anti-tumor immune response. Upon stimulation by tumor antigens and other immune cells, CD8^+^ T cells undergo metabolic reprogramming through signaling pathways. Metabolites or metabolic checkpoints induce epigenetic changes in key genes, altering the differentiation and effector function of CD8^+^ T cells. This review first elaborates on the anti-tumor functional characteristics and metabolic profiles of CD8^+^ T cells at different stages of differentiation in the TME. Then we focus on the roles of key metabolites and metabolic checkpoints in shaping CD8^+^ T cell differentiation, functionality, and immune responses, specifically through glucose, lipid, and amino acid metabolism. Finally, we discuss the reasons for heterogeneity in the effects of metabolic checkpoints on CD8^+^ T cells and explore potential clinical applications of metabolic checkpoints in treatment. Understanding the correlation between CD8^+^ T cell metabolism and anti-tumor immunotherapy may facilitate the development of new strategies to enhance the efficacy of CD8^+^ T cell-based cancer treatments.

## Introduction

Tumor development is accompanied by changes in many metabolic pathways 1. In 1927, the German physiologist Otto H. Warburg first observed that cancer cells grow much faster than normal cells due to differences in energy acquisition. While normal cells typically obtain energy through aerobic respiration, cancer cells favor glycolysis, known as the “Warburg effect” which is considered a sign of malignancy 2. Recent studies have revealed that, in addition to aerobic glycolysis, highly metabolically active tumor cells also rely on diverse metabolic pathways such as increased oxidative phosphorylation, altered fatty acid oxidation and synthesis, and amino acid metabolism to produce large amounts of lactate and other potentially toxic metabolites 1. At the same time, the uncontrolled proliferation of tumor cells and disorganized vasculature within tumors lead to increased oxygen consumption and insufficient oxygen supply, creating a microenvironment characterized by acidity, hypoxia, and nutrient depletion. This microenvironment promotes tumor cell growth and metastasis while suppressing immune cell growth and impairing their immune surveillance functions 3. As a result, cancer is increasingly recognized as a metabolic disorder 1, 4, 5.

In fact, alongside the metabolic reprogramming of tumor cells, other immune cells also undergo adaptive metabolic changes. There is often metabolic competition between these infiltrating immune cells and tumor cells, leading to the formation of a microenvironment characterized by the metabolic interactions and “cross-talk” between tumor and immune cell metabolism, which is referred to as the “tumor microenvironment” (TME). Immune cells and tumor cells not only passively adapt to their surroundings but also actively shape the environment by producing metabolic products such as lactate. These metabolites influence the survival and function of neighboring cells in the same environment, thereby establishing bidirectional metabolic communication between immune cells and their surrounding environment. Based on the aforementioned research background and drawing inspiration from immunologists' nomenclature for immune checkpoints, metabolic checkpoints are currently defined as trigger targets that alter metabolic patterns in different immune cell populations. These metabolic checkpoints have potential influence on the functional differentiation and fate determination of immune cells. The distinctions and interrelationship between immune checkpoints and metabolic checkpoints are presented in Table 1 6-8.

T cells are the core mediators of anti-tumor immunity 5. Especially, CD8^+^ cytotoxic T lymphocytes (CTLs) are considered as the main effector cells involved in the anti-tumor immune process. Long-lived memory CD8^+^ T cells are regarded as a convenient biomarker for assessing the status and efficacy of CD8^+^ T cells within the immune system. Exhausted CD8^+^ T cells gradually lose their original effector functions, leading to the immune system's inability to effectively clear tumor cells from the body 9. Increasing evidence suggests that the metabolic adaptability of CD8^+^ T cells determines their function and fate 10. This review will discuss the metabolic characteristics of CD8^+^ T cells at different differentiation stages and the existing research progress on CD8^+^ T cell metabolic checkpoints in glucose, lipid, and amino acid metabolism in the TME.

## Anti-tumor functions of CD8^+^ T cells at different differentiation stages

Naïve lymphocytes enter the peripheral blood from peripheral lymphoid tissues. Upon stimulation by tumor antigens and co-stimulation, naïve lymphocytes exit their “quiescent phase” 11 and develop into various functional T cell subpopulations 12. Some differentiate into effector lymphocytes, such as effector CD8^+^ T cells, which can exert anti-tumor functions by secreting cytotoxic factors and inducing apoptosis in target cells through direct binding to the Fas cell surface death receptor (Fas). Others differentiate into memory lymphocytes, including memory CD8^+^ T cells 13, which can be categorized into three main subgroups based on surface markers and migration patterns: central memory T (Tcm) cells, effector memory T (Tem) cells, and non-circulating tissue-resident memory T (Trm) cells 14. Stem cell memory T (Tscm) cells can self-renew in lymph nodes and differentiate into Tem and Trm cells. Trm cells can migrate to the TME through circulation, secreting pro-inflammatory cytokines such as interferon-gamma (IFN-γ) and tumor necrosis factor-alpha (TNF-α) to recruit and activate tumor-specific CD8⁺ T cells. Trm cells can also activate dendritic cells, increasing the number of tumor-specific CD8^+^ T cells, thereby inhibiting tumor growth and dissemination 15-17.

**Figure 1:**
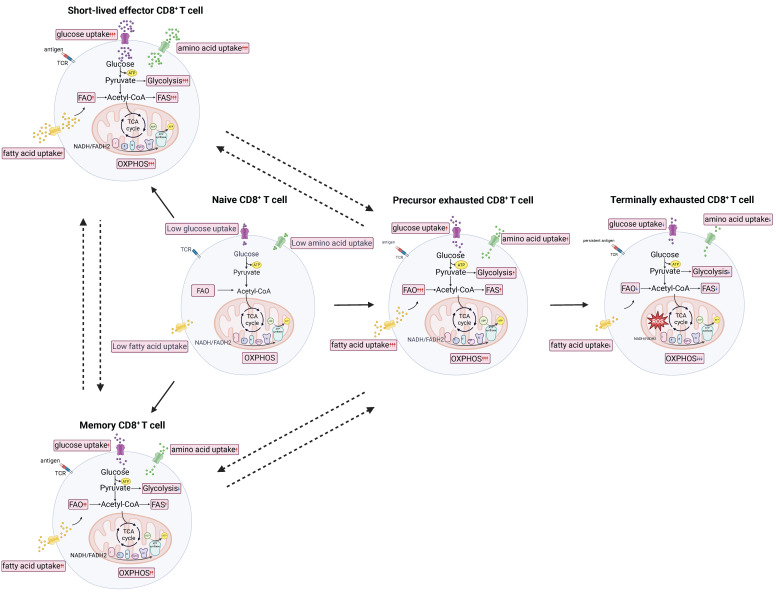
**Metabolic characteristics of CD8^+^ T cells at different stages.** Naive CD8^+^ T cells are able to differentiate into three subtypes under different conditions, namely short-lived effector T (Teff) cells, memory T (Tm) cells, and precursor exhausted T (Tpex) cells. In addition, the three subtypes of CD8^+^ T cells can directly or indirectly transform into each other under specific conditions. Among them, Tpex cells will further differentiate into terminally exhausted T (Ttex) cells under persistent stimulation of tumor antigens, and this process is irreversible. ATP: adenosine triphosphate; FADH2: flavin adenine dinucleotide dihydrogen; FAO: fatty acid oxidation; FAS: fatty acid synthesis; NADH: nicotinamide adenine dinucleotide hydride; OXPHOS: oxidative phosphorylation; ROS: reactive oxygen species; TCA: tricarboxylic acid; TCR: T cell receptor.

Persistent antigen stimulation causes some antigen-specific CD8^+^ T cells to lose effector functions, leading to the fading of memory T cell characteristics and eventual progression to exhaustion known as exhausted T (Tex) cells. In the context of CD8^+^ T cell exhaustion, precursor exhausted T (Tpex) and terminally exhausted T (Ttex) T cells are often distinguished by their expression of T cell factor 1 (TCF1) and T-cell immunoglobulin and mucin-domain containing-3 (TIM-3). Early studies reported that Tpex cells (PD-1⁺ TCF1⁺ TIM-3⁻) can undergo significant expansion in response to anti-PD-L1 treatment, whereas Ttex cells (PD-1⁺ TCF1⁻ TIM-3⁺) are largely unresponsive 18. Furthermore, recent studies have found that Tpex cells can differentiate into effector T (Teff) cells and exert anti-tumor effects under certain conditions, whereas Ttex cells are irreversible 19 (Figure 1). According to the report, Tpex cells can be reversed by immune checkpoint blockers (ICBs) such as PD-1 antibodies, restoring a certain level of proliferation and immune response. In contrast, Ttex cells continue to express inhibitory receptors characteristically even when antigen stimulation ceases, displaying very low proliferative capacity and effector functions, and they cannot regain their anti-tumor or anti-viral efficacy through PD-1 inhibitor treatment 18.

## Metabolic characteristics of CD8^+^ T cells at different differentiation stages

When the immune system is stable, CD8^+^ T cells remain in a quiescent state. However, when T cells are stimulated by tumor antigens, they are rapidly activated 20. Researches have shown that there are significant differences in the metabolic pathways between the quiescent and activated states of T cells 12.

Naïve CD8^+^ T cells that have not been stimulated by antigens are in a quiescent state due to the inhibition of mechanistic target of rapamycin complex 1 (mTORC1) activity, which in turn suppresses the production of reactive oxygen species (ROS). As a result, these cells exhibit low overall metabolic, transcriptional, and translational activity, with reduced mitochondrial activity and glucose uptake. They primarily generate adenosine triphosphate (ATP) through mitochondrial oxidative phosphorylation (OXPHOS) and fatty acid oxidation (FAO) 20. After encountering antigens, naïve T cells begin to activate. Within the first 6 hours of activation, aerobic glycolysis is upregulated. Within 24 hours, OXPHOS is upregulated, along with the tricarboxylic acid (TCA) cycle, pentose phosphate pathway (PPP), lipid synthesis, and amino acid metabolism. Eventually, naïve CD8^+^ T cells shift to a metabolism primarily driven by aerobic glycolysis to sustain their effector functions 21. Interestingly, the activity and function of effector CD8^+^ T cells largely depend on steroid metabolism. Cholesterol synthesis plays a key role in T cell activation and proliferation 22. When Teff cells and tumor cells compete for glucose at a disadvantage, Teff cells may actively adjust their metabolic mode to fatty acid energy supply, activate fatty acid metabolic pathways, and maintain cellular energy supply 23. However, studies have shown that despite the accumulation of lipid droplets of CD8^+^ T cells in tumors, the killing power of Teff cells is not strong, indicating that fatty acid energy supply is not always beneficial for the anti-tumor ability of Teff cells 24. Memory CD8^+^ T cells exhibit increased FAO and OXPHOS at the overall metabolic level, relying on FAO for long-term persistence and rapid proliferation upon antigen re-exposure 25. Notably, the metabolic characteristics of different subtypes of memory CD8^+^ T cells vary slightly. Tcm cells show increased levels of OXPHOS, Tem cells exhibit increased glycolysis, and Trm cells primarily rely on fatty acid β-oxidation and mitochondrial oxidative metabolism 20. Another study suggests that inhibiting glucose metabolism in CD8^+^ T cells can enhance their memory function and anti-tumor immunity 26. During prolonged antigen exposure, the normal metabolic regulation of CD8^+^ T cells is disrupted, leading to the formation of Tex cells. Tex cells exhibit significant changes in mitochondrial abundance, dynamics, membrane potential, OXPHOS, and the production of mitochondrial ROS. Specifically, their metabolic function is characterized by reduced glucose uptake, along with decreased mitochondrial quality and function 27. At this point, the glycolysis and OXPHOS levels of Tex cells decrease 28.

Overall, CD8⁺ T cells display distinct metabolic characteristics across different differentiation stages and subtypes, and metabolic regulation is a key determinant of their differentiation and functional fate 29 (Figure 1). For example, memory CD8⁺ T cells exhibit enhanced OXPHOS and FAO, whereas inhibition of glycolysis promotes the memory phenotype and function 26, 28.

## Research progress on metabolic checkpoints of CD8^+^ T cells in the TME

**Figure 2:**
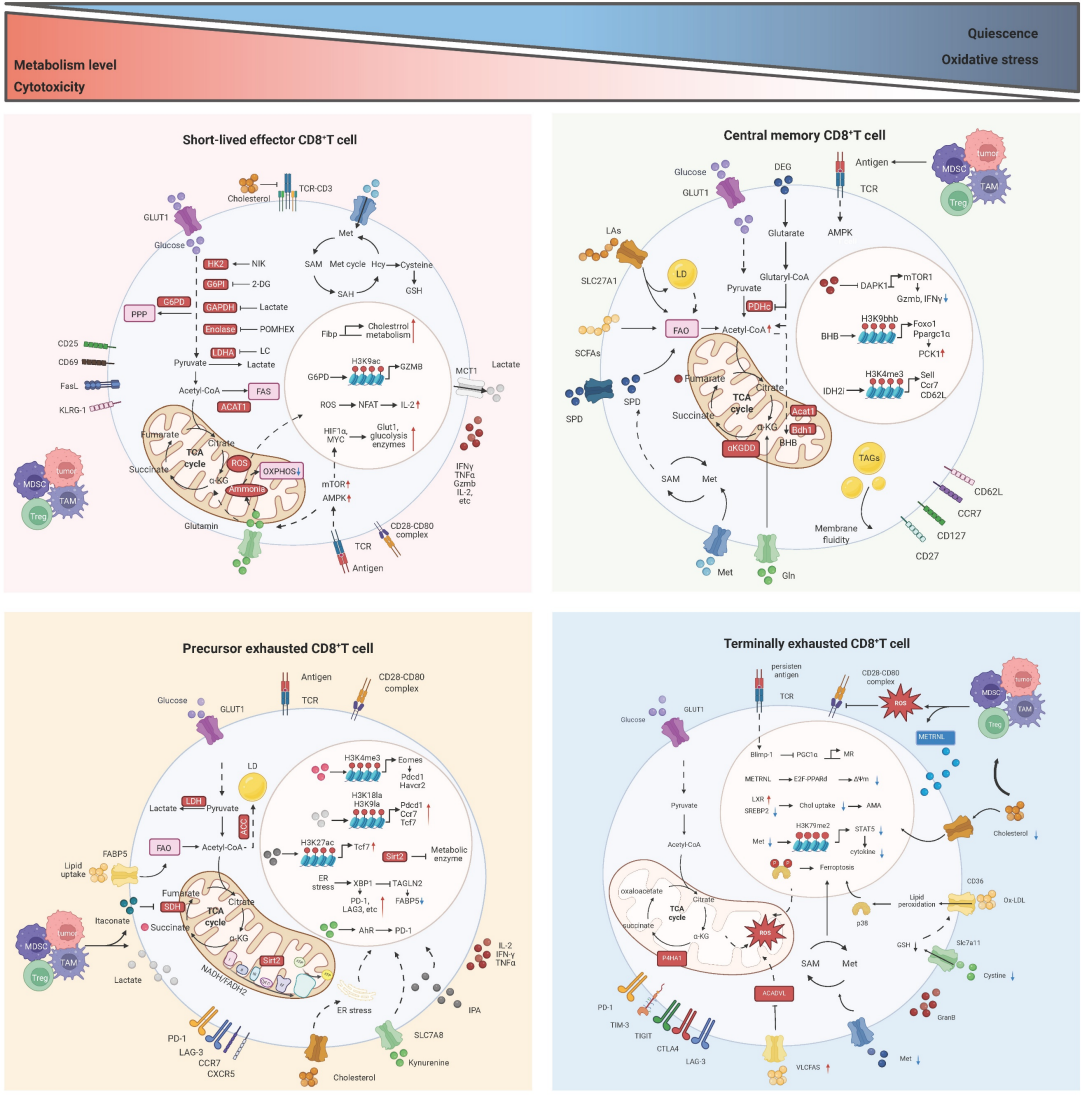
** The main metabolic checkpoint regulatory mechanism of CD8^+^ T cells in TME.** At the overall metabolic level, CD8^+^ effector T (Teff) cells and CD8^+^ precursor exhausted T (Tpex) cells exhibit more active metabolism and stronger cytotoxicity. Conversely, CD8^+^ memory T (Tm) cells and CD8^+^ terminally exhausted T (Ttex) cells demonstrate the opposite. ACADVL: acyl-CoA dehydrogenase very long chain; ACC1: acetyl-CoA carboxylase 1; ACAT1: acetyl coenzyme A acetyltransferase 1; AhR: aryl hydrocarbon receptor; AMPK: AMP-activated protein kinase; AMA: autophagy mediated apoptosis; α-KG: α-ketoglutarate; ATP: adenosine triphosphate; Bdh1: D-β-hydroxybutyrate dehydrogenase; BHB: β-hydroxybutyrate; CCR7: C-C motif chemokine receptor 7; Chol: cholesterol; CTLA4: cytotoxic T-lymphocyte associated protein 4; CXCR5: C-X-C motif chemokine receptor 5; DAPK1: death associated protein kinase 1; DEG: diethyl 2-hydroxyglutarate; Eomes: eomesodermin; ER: endoplasmic reticulum; FABP5: fatty acid binding protein 5; FA: fatty acid; FAO: fatty acid oxidation; FAS: fatty acid synthesis; FasL: Fas ligand; Fibp: fibroblast growth factor 1 intracellular binding protein; Foxo1: forkhead box O1; G6PD: glucose-6-phosphate dehydrogenase; G6PI: glucose-6-phosphate isomerase; GAPDH: glyceraldehyde-3-phosphate dehydrogenase; GLUT1: glucose transporter 1; GSH: glutathione; Gln: glutamine; Havcr2: hepatitis A virus cellular receptor 2; Hcy: homocysteine; HK2: hexokinase 2; IDH2: isocitrate dehydrogenase 2; IPA: indole-3-propionic acid; KLRG1: killer cell lectin-like receptor subfamily G member 1; LAG3: lymphocyte activation gene-3 protein; LA: linoleic acid; LC: lithium carbonate; LCFAs: long-chain fatty acid; LD: lipid droplet; LDHA: lactic dehydrogenase; MCT1: monocarboxylate transporter 1; MDSC: myeloid derived suppressor cells; METRNL: meteorin-like protein; Met: methionine; MR: mitochondrial reprogramming; mTOR: mechanistic target of rapamycin; NADH: nicotinamide adenine dinucleotide hydride; NFAT: nuclear factor of activated T cells‌; OXPHOS: oxidative phosphorylation; Ox-LDL: oxidized low-density lipoprotein; P4HA1: prolyl 4-hydroxylase 1; PCK1: phosphoenolpyruvate carboxy kinase 1; PDHc: pyruvate dehydrogenase complex; Pdcd1: programmed cell death 1; POMHEX: a cell-permeable pivaloyloxymethyl (POM) pro-drug of HEX; PPARδ: peroxisome proliferator activated receptor delta; PPP: pentose phosphate pathway; ROS: reactive oxygen species; SAH: s-adenosyl-L-homocysteine; SAM: s-adenosylmethionine; SCFA: short-chain fatty acid; SDH: succinate dehydrogenase; Sell: selectin L; Sirt2: sirtuin 2; SLC27A1: solute carrier family 27 member 1; SLC7A11: solute carrier family 7 member 11; SLC7A8: solute carrier family 7 member 8; SPD: spermidine; SREBP2: sterol-regulatory element binding protein 2; TAGLN2: transgelin 2; TAGs: triacylglycerol; TAM: tumor associated macrophages; TCA: tricarboxylic acid; Tcf7: transcription factor 7; TCR: T cell receptor; TIGIT: T cell immunoreceptor with Ig and ITIM domains; TIM3: T-cell immunoglobulin and mucin-domain containing-3; TME: tumor microenvironment; Treg: regulatory T cell; VLCFAs: very long chain fatty acids; XBP1: X-box binding protein 1; ∆Ψm: mitochondrial membrane potential.

The metabolic reprogramming of tumor-infiltrating CD8^+^ T cells is influenced by the complex and variable TME. Similar to other cells, T cells engage in continuous communication with their surrounding environment and can appropriately interpret and respond to environmental signals, including metabolic conditions 29. Since immune cells and tumor cells are exposed to similar TME conditions, they share many metabolic characteristics. At the same time, immune cells and tumor cells compete for nutrients, and nutrient deprivation in T cells can impair their proliferation, differentiation, or function 9. Therefore, in the harsh environment of the TME, T cells must sense extracellular nutrients and maintain flexible intracellular metabolic pathways that align with nutrient utilization to support T cell survival, subset differentiation, energy supply, and biosynthesis. Metabolic checkpoints can influence the functional differentiation and fate determination of CD8^+^ T cells. Certain metabolites and intermediate metabolites can serve as novel signaling molecules, exerting a profound impact on immune regulation; metabolic enzymes, acting as immune mediators, can catalyze specific enzymatic reactions; and transporters, functioning as passageways for the transport of nutrients and metabolites, can regulate the metabolic pathways of CD8^+^ T cells 30. Targeting metabolic checkpoints, encompassing specific nutrients or intermediate metabolites, key enzymes, and transporters, may potentially bolster the anti-tumor capabilities of CD8^+^ T cells. Next, the metabolic checkpoints existing in CD8^+^ T cells and their roles and research status will be elaborated from the three main modules of glucose metabolism, lipid metabolism and amino acid metabolism (Figure 2). Table 2 summarizes the clinical trials of current strategies targeting CD8⁺ T cell metabolic dysfunction in tumors.

### Glucose metabolism

Glucose metabolism in T cells mainly includes four pathways: glycolysis, PPP, OXPHOS, and the TCA cycle. Glycolysis and PPP primarily occur in the cytoplasm, as well as OXPHOS and the TCA cycle mainly take place in the mitochondria.

#### Glycolysis

Following T cell activation, aerobic glycolysis is upregulated, with glucose predominantly transported into cells via glucose transporter 1 (GLUT1) 31. The glucose is then broken down into pyruvate through a series of enzymatic reactions (such as those catalyzed by hexokinase 2, enolase, etc.). Pyruvate can further be reduced to lactate by lactate dehydrogenase (LDH), producing a small amount of ATP to provide energy for the T cells 5, 32. Not surprisingly, these key enzymes and intermediate metabolites directly and indirectly regulate T cell proliferation, differentiation, migration, and anti-tumor functions 33.

According to the report, in the acute lymphoblastic leukemia (ALL) mouse model, overexpression of GLUT1 can promote the formation of T cell memory and prolong survival by enhancing glycolysis and mitochondrial respiration pathways 31. Consistent with this view, hypoxia-inducible factor HIFα and myelocytomatosis oncogene (MYC) can promote glycolysis by increasing the expression of glucose transporter proteins on the surface of T cells, thereby enhancing lymphocyte infiltration and cytotoxic function 5, 34, 35. Additionally, it has been reported that ectopic expression of NF-κB-inducing kinase (NIK) promotes glycolysis by preventing the autophagic degradation of the rate-limiting enzyme hexokinase 2, which significantly enhances the effector function of CD8^+^ T cells and improves the efficacy of T cell adoptive therapy 36. In line with this report, recent studies have shown that treating CD8^+^ T cells with 2-deoxyglucose (2-DG) (a glucose analogue that inhibits hexokinase and glucose-6-phosphate isomerase) and POMHEX (enolase inhibitor) to inhibit glycolysis results in reduced activation capacity of CD8^+^ T cells 37.

The effect of lactate on CD8^+^ T cell function is heterogeneous. Hypoxia induced aerobic glycolysis in tumor cells is accompanied by an increase in lactate secretion. Excessive lactate is discharged into the TME by tumor cells, resulting in an acidic microenvironment 38. For one thing, high concentrations of lactate in the tissue microenvironment directly inhibit effector functions, including the production of IFN-γ and TNF-α, as well as the infiltration of CD8^+^ T cells 39, 40. In addition, lactate can indirectly inhibit the proliferation, survival and migration of effector CD8^+^ T cells by suppressing the activity of metabolic enzymes and signal transduction. For example, lactate hinders the proliferation of effector CD8^+^ T cells by impeding the activities of glyceraldehyde 3-phosphate dehydrogenase (GAPDH) and phosphoglycerate dehydrogenase (PHGDH) 41. Besides, lactate can inhibit autocrine signal transduction in cytotoxic CD8^+^ T cells by reducing pyruvate carboxylase (PC) activity, succinate secretion and succinate receptor 1 (SUCNR1) activation 42. Under hypoxic conditions, CD8^+^ T cells generate pyruvate through glycolysis, which is subsequently reduced to lactate by LDH. This process concomitantly oxidizes nicotinamide adenine dinucleotide hydride (NADH) to nicotinamide adenine dinucleotide (NAD^+^), thereby sustaining the continuous progression of glycolysis. Consequently, LDH plays a key role in regulating cytokine mediated differentiation of CD8^+^ T cells. Inhibition of LDH promotes the entry of pyruvate into the TCA cycle, thereby suppressing terminal effector and exhaustion of CD8^+^ T cells. This effect is associated with reduced expression of nuclear receptor 4As (NR4As), PR domain zinc finger protein 1 (PRDM1), X-box binding protein 1 (XBP1), as well as exhaustion markers including lymphocyte activation gene-3 protein (LAG-3), PD-1, 2B4 (CD244), and TIM-3. LDH inhibition combined with IL-21 can promote the formation of Tscm cells, thus producing a more profound anti-tumor response and prolonging the survival time of the host 43. For another thing, lactate derived from aerobic glycolysis within activated CD8⁺ T cells exerts distinct immunomodulatory effects. The latest research demonstrates that LDH-mediated endogenous lactate promotes the stem cell-like phenotype of CD8⁺ T cells and enhances anti-tumor immunity. Mechanistically, lactate upregulates Tcf7 expression in CD8⁺ T cells by inducing histone H3 lysine 27 acetylation (H3K27ac) and histone H3 lysine 9 lactylation (H3K9la) enrichment, which leads to an increased proportion of TCF1⁺ CD8⁺ T cells 37, 44. *In vitro* experiments showed that inhibition of lactate production by the LDH inhibitor A resulted in an increased cancer cell-to-CD8⁺ T cell ratio and a decreased proportion of apoptotic cancer cells, indicating reduced T cell-mediated cytotoxicity 37. These results suggest that the intervention of key enzymes and metabolic intermediates in the process of aerobic glycolysis may effectively interfere with the proliferation, differentiation, migration and cytotoxic function of CD8^+^ T cells, so as to enhance the anti-tumor function of CD8^+^ T cells.

#### Pentose phosphate pathway (PPP)

The PPP is an alternative pathway of glucose metabolism. In this pathway, cytosolic glucose-6-phosphate is converted into phosphoribose and nicotinamide adenine dinucleotide phosphate hydride (NADPH) through a series of enzymatic reactions initiated by glucose-6-phosphate dehydrogenase (G6PD), providing substrates for nucleotide biosynthesis and hydrogen donors for various metabolic reactions. It is worth noting that activation of the rate limiting enzyme G6PD in the PPP can increase the production of of acetyl-CoA, leading to enhanced deposition of H3K9 acetylation (H3K9ac) at the granzyme B (Gzmb) promoter and increased expression of Gzmb in tumor-specific CTLs. This process converts them from a Gzmb^lo^ phenotype to a Gzmb^hi^ phenotype, thereby enhancing CTL-mediated tumor lysis activity. Accordingly, *in vivo*, G6PD activation is accompanied by an expansion of Gzmb⁺ tumor-specific CTLs and attenuation of tumor growth in tumor-bearing mice 45.

#### Tricarboxylic acid (TCA) cycle

The TCA cycle plays a crucial role in CD8^+^ T cell metabolism. Both naïve and memory CD8^+^ T cells perform complete pyruvate oxidation via the TCA cycle, generating NADH and flavin adenine dinucleotide dihydrogen (FADH2). These metabolites not only provide substrates for OXPHOS but also regulate various metabolites with metabolic and signaling functions, such as acetyl-CoA, citrate, fumarate, α-ketoglutarate (α-KG), and succinate 46. It is not surprising that many of these metabolic intermediates and the key enzymes involved also have direct or indirect immunoregulatory roles.

Isocitrate dehydrogenase (IDH) catalyzes the conversion of isocitrate to oxalosuccinate in the TCA cycle, which is further decarboxylated to produce α-KG. Recently, a screening of compounds related to leukemia revealed that the FDA-approved IDH2 inhibitor enasidenib enhances the formation of memory chimeric antigen receptor (CAR)-T cells and sustains anti-leukemia cytotoxicity *in vivo*. Further research showed that IDH2 inhibits glucose utilization via the PPP, hindering the metabolic adaptability of CAR-T cells, while the PPP exacerbates oxidative stress. Additionally, IDH2 limits cytosolic acetyl-CoA levels, preventing histone acetylation that promotes memory cell formation 47.

Succinate dehydrogenase (SDH) is a key mitochondrial enzyme that catalyzes the dehydrogenation of succinate to fumarate in the TCA cycle, producing fumarate and FADH2. In the TME, itaconate has been reported to inhibit SDH activity in CD8⁺ T cells, leading to succinate accumulation that promotes histone H3 lysine 4 trimethylation (H3K4me3)-mediated transcription of Eomesodermin (Eomes) and the subsequent upregulation of exhaustion markers PD-1 and TIM-3, thereby inducing CD8^+^ T cell exhaustion 48. However, another study demonstrated that under persistent antigen stimulation, succinate treatment enhances glycolysis and the TCA cycle metabolism in CD8^+^ T cells, upregulates multiple mitophagy-related genes, and thereby promotes CD8^+^ T cell survival and expansion. Concurrently, by reducing the intracellular α-KG/succinate ratio, it modulates the activity of DNA and histone demethylases, leading to increased expression of genes such as Tcf7, Id3, and Batf3, which drives T cells toward a stem-like state. Together, these studies highlight that succinate can exert divergent effects on CD8⁺ T cell fate, depending on their subset and the TME context 49**.**

2-Hydroxyglutarate (2-HG) structurally resembles the TCA cycle intermediate α-KG. As a competitive inhibitor of α-KG, 2-HG can inhibit the activity of various α-KG-dependent dioxygenases (α-KGDDs) and regulate multiple metabolic reactions in CD8^+^ T cells. This includes inducing succinylation of pyruvate dehydrogenase E2 (PDHE2), which in turn increases glycolysis rates and maintains or enhances mitochondrial oxidative activity. Analysis of memory populations in mouse and human CD8^+^ T cells treated with diethyl 2-hydroxyglutarate (DEG), a cell-permeable form of 2-HG, revealed that DEG increased the Tcm-cell population in both mouse and human differentiated CD8^+^ T cells, while reducing the Tem-cell population and enhancing cytotoxicity against target cells. Specifically, this was reflected in increased expression of cytotoxic effector molecules, such as GZMB, and reduced expression of exhaustion markers like thymocyte selection-associated high mobility group box protein (TOX), TIM-3, and LAG-3 50.

Prolyl 4-hydroxylase 1 (P4HA1) is an α-KG dependent metabolic enzyme. In the human CAR-T cell model, P4HA1 accumulates in mitochondria and disrupts the TCA cycle through abnormal metabolism of α-KG and succinic acid, damaging mitochondrial adaptation while inhibiting CD8^+^ Tpex cell expansion. Targeting P4HA1 can enhance adoptive and endogenous TCF1^+^CD8^+^ T progenitor cell expansion, while alleviating the development of terminal exhaustion in tumors and tumor draining lymph nodes, thereby achieving significant and persistent systemic anti-cancer immunity 51.

Fumarate, an intermediate metabolite in the TCA cycle, plays a critical role in regulating T cell function. It has been reported that fumarate accumulates in T cell mitochondria due to various factors, such as the deletion of complement C1qb 52, the deletion of malic enzyme 2 (ME2) 53, or the uptake of fumarate secreted by tumor cells into CD8^+^ T cells 54. This accumulation can suppress T cell proliferation, differentiation, and function through multiple mechanisms. Earlier studies suggested that, similar to 2-HG, fumarate is also a competitive inhibitor of α-KG-dependent dioxygenases, inhibiting CD8^+^ T cell differentiation and function by reversibly inhibiting dioxygenases involved in epigenetic signaling 52. Recent research has added another regulatory pathway, showing that fumarate can directly bind to death-associated protein kinase 1 (DAPK1) and inhibit its kinase activity by competing with ATP binding. This suppresses the downstream tuberous sclerosis complex 2 (TSC2)-mTORC1 signaling pathway, leading to reduced activation, effector function, and anti-tumor activity of CD8^+^ T cells 53.

#### Oxidative phosphorylation (OXPHOS)

Tumor cells predominantly utilize aerobic glycolysis for energy generation, resulting in glucose depletion in the TME. This glucose shortage reduces mTOR activity in CD8^+^ T cells, thereby forcing these cells to switch to OXPHOS as their main energy needs 55. ROS are produced during mitochondrial OXPHOS. Initially, ROS were simply considered harmful byproducts of cellular metabolism. However, recent research has revealed that ROS have a “double-edged sword” effect. On the one hand, a moderate amount of ROS is essential for CD8^+^ T cell activation; on the other hand, excessive ROS production can lead to oxidative stress, causing CD8^+^ T cell damage or driving them into a state of terminal exhaustion, which induces immune-suppressive effects 56. A comprehensive understanding of this graded dose effect of ROS on immune cells is crucial for developing effective strategies to improve cancer immunotherapy.

Upon stimulation of the T cell receptor (TCR), mitochondrial oxidative phosphorylation in T cells is activated, leading to the generation of a moderate amount of ROS associated with active metabolism. This metabolic shift enhances CD8^+^ T cell proliferation and interleukin production 57. Similarly, another viewpoint suggests that moderate ROS stress can activate nuclear factor of activated T cells (NFAT) and promote IL-2 secretion 58. Additionally, ROS can rapidly upregulate the aryl hydrocarbon receptor (AhR) in effector CD8^+^ T cells, programming them for differentiation into memory CD8^+^ T cells 59.

Under hypoxic conditions, continuous tumor antigen stimulation of CD8^+^ T cells activates the transcriptional repressor Blimp-1 (encoded by Prdm1), which inhibits the expression of peroxisome proliferator-activated receptor-gamma coactivator (PGC)-1α, a transcriptional coactivator that coordinates mitochondrial biogenesis and antioxidant activity. This leads to impaired mitochondrial reprogramming and consequent mitochondrial dysfunction, which in turn promotes ROS production through multiple mechanisms. Examination of endogenous B16 tumor-infiltrating lymphocytes (TILs) revealed that CD8^+^ Tex cells have significantly higher levels of mitochondrial ROS (mtROS). Continuous activation under *in vitro* hypoxic conditions also produces elevated levels of mtROS, suggesting that ROS may be a driving factor in T cell exhaustion 60. Recent studies show that meteorin-like (METRNL) protein, secreted by CD8^+^ T cells during repeated stimulation, can cause ROS accumulation, DNA damage, and apoptosis of CD8^+^ T cells 61. Moreover, it has been reported that metformin, by reducing ROS accumulation in CD8^+^ T cells, prevents mitochondrial dysfunction and oxidative DNA damage, thereby directly improving CD8^+^ T cell survival and function 56. In addition to ROS generated by CD8^+^ T cells themselves, cancer cells and immune-suppressive cells, including myeloid-derived suppressor cells (MDSCs) and regulatory T cells (Tregs), can release high levels of ROS into the TME, resulting in the suppression of T cell responses 62.

NAD^+^ accepts hydrogen atoms from the TCA cycle and the mitochondrial respiratory chain, forming the reduced NADH, which is a key step in intracellular oxidative phosphorylation. Sirtuin 2 (Sirt2) is a NAD^+^ dependent deacetylase. Experimental evidence shows that Sirt2 can induce the deacetylation of various metabolic enzymes in CD8^+^ T cells, leading to a downregulation of their activity, which results in a reduction of both aerobic glycolysis and OXPHOS. In human TILs isolated from non-small cell lung cancer (NSCLC) patient samples treated with the Sirt2 inhibitor AGK2, an increase in aerobic glycolysis, OXPHOS, and IFN-γ production was observed. Therefore, inhibition of Sirt2 may serve as a promising intervention to enhance the metabolic adaptability of tumor-reactive CD8^+^ T cells in the context of cell therapy 63.

In summary, glucose metabolism plays a crucial role in CD8^+^ T cell function, affecting various aspects including CD8^+^ T cell proliferation, activation, differentiation, and effector functions. At the same time, metabolites produced during glucose metabolism, such as ROS, can have a dual role. Interestingly, in CD8^+^ T cells, glycolysis and OXPHOS act like two sides of the same coin, meaning that the inhibition of glycolysis is accompanied by an increase in OXPHOS. For instance, in mouse models, asparagine restriction, L-arginine supplementation, and transient glucose restriction (TGR) all facilitate a metabolic pathway shift from glycolysis to OXPHOS in activated T cells, thereby enhancing their anti-tumor activity 64-66. Glucose related metabolism checkpoints influence CD8^+^ T cell proliferation, activation, differentiation, and effector functions by modulating key transcription factors through epigenetic modifications, affecting key molecules in signaling pathways, or directly influencing one or more metabolic cycles. These metabolic processes are integral to the regulation of CD8^+^ T cell responses and immune function.

### Lipid metabolism

Lipid metabolism plays a pivotal role in CD8^+^ T cell function and fate and encompasses multiple dimensions, including extracellular lipid signaling, intracellular lipid metabolic reprogramming, membrane lipid regulation, and lipid-mediated regulation of gene transcription. These lipid metabolic pathways are essential not only for maintaining cellular energy balance but also for supporting the differentiation and functional activation of CD8^+^ T cells, which can be modulated by changes in lipid availability and utilization. Among these metabolic pathways, fatty acid oxidation (FAO), fatty acid synthesis (FAS), fatty acid transport, ferroptosis and cholesterol metabolism, are elaborated in detail due to their crucial roles in CD8^+^ T cells.

#### Fatty acid oxidation (FAO)

In the TME, T cells must metabolize lipids through mitochondrial fatty acid oxidation to provide energy under nutrient stress conditions, and FAO-enriched CD8^+^ T cells have been shown to be more proficient at controlling cancer 67. FAO breaks down fatty acids into acetyl-CoA, which then enters the TCA cycle via β-oxidation, ultimately producing energy and carbon dioxide. The substrates involved in lipid metabolism include short-chain fatty acids (SCFAs) and long-chain fatty acids (LCFAs). It has been reported that in germ-free (GF) mice, butyrate, a SCFA produced by gut microbiota fermentation, can promote the differentiation of memory CD8^+^ T cells by enhancing the uptake and oxidation of fatty acids in activated CD8^+^ T cells 68, 69. LCFAs can serve as substrates for FAO. Under the catalysis of fatty acyl-CoA synthetase, LCFAs are converted into acyl-CoA, which is subsequently broken down into acetyl-CoA 69. However, studies have found that in a pancreatic ductal adenocarcinoma (PDA) mouse model, CD8^+^ T cells gradually accumulate specific LCFAs, particularly very long-chain fatty acids (VLCFAs), triggering a major transcriptional reprogramming of lipid metabolism pathways. This leads to reduced fatty acid catabolism and impaired mitochondrial function in CD8^+^ T cells. Specifically, in the pancreas, CD8^+^ T cells exhibit downregulation of very long-chain acyl-CoA dehydrogenase (VLCAD), which exacerbates the accumulation of LCFAs and VLCFAs, both of which contribute to lipotoxicity. Therefore, forced expression of acyl-CoA dehydrogenase very long chain (ACADVL, which encodes VLCAD) in tumor-specific T cells and metabolic reprogramming can enhance T cell survival and persistence within tumors in a PDA mouse model, offering a potential therapeutic target for PDA immunotherapy 70. Supporting this notion, independent research further revealed that genetic ablation of the VLCFA elongase Elovl1 boosted CD8⁺ T cell-mediated tumor control in several models, including PDA and melanoma, especially in combination with anti-PD-1 blockade. Thus, the findings imply that a therapeutic strategy focused on disrupting the specific pathways leading to toxic VLCFA accumulation, rather than on globally stimulating or suppressing FAO, may offer a more universally relevant metabolic intervention to enhance immunotherapy 71.

Acetyl-CoA plays a crucial role in lipid metabolism, acting as both a metabolite of fatty acid oxidation and a substrate for fatty acid synthesis, while also serving as a central metabolic hub that integrates diverse biosynthetic and catabolic processes. Furthermore, the acetyl-CoA serves as an essential substrate for histone acetylation and IFN-γ production, thereby enhancing the metabolism and function of CD8^+^ T cells within the TME 72. However, low levels of TCA cycle activity (e.g. low oxaloacetate levels) can drive acetyl-CoA toward the ketogenic pathway, leading to the production of β-hydroxybutyrate (BHB). This, in turn, indirectly upregulates the expression of phosphoenolpyruvate carboxy kinase 1 (PCK1), a rate-limiting enzyme in gluconeogenesis, thus regulating the formation and maintenance of CD8^+^ memory T cells 72.

It is noteworthy that long-chain polyunsaturated fatty acid arachidonic acid (AA) and IFN-γ secreted by CD8^+^ T cells synergistically induce tumor cell ferroptosis through acyl-CoA synthetase long-chain family member 4 (ACSL4) 73. Therefore, targeting the ACSL4 pathway represents a potential anticancer strategy. Additionally, tumor-derived prostaglandin E2 (PGE2), an enzymatic metabolite of AA, can restrict the proliferation and effector differentiation of TCF1^+^CD8^+^ T cells via the EP2 and EP4 (EP2/EP4) receptor signaling pathways in CD8^+^ T cells, thereby promoting cancer immune evasion 74. The PGE2-EP2/EP4 axis may serve as a molecular target for achieving cancer immune control. Beyond acyl-CoA synthetases, another key enzyme in fatty acid β-oxidation is carnitine palmitoyltransferase 1 (CPT1). In effector CD8^+^ T cells, activated STAT3 binds to CPT1B, driving increased FAO, suppressing glycolysis and IFN-γ secretion, and ultimately facilitating breast cancer progression 23.

#### Fatty acid synthesis (FAS)

Fatty acid synthesis utilizes acetyl-CoA to generate new lipids, such as triglycerides, cholesterol, ketone bodies, and phospholipids. This process requires substantial amounts of energy and raw materials. Cytosolic fatty acid synthesis directly opposes mitochondrial fatty acid oxidation and is controlled by acetyl-CoA carboxylase (ACC), the rate-limiting enzyme in de novo fatty acid synthesis. However, studies have shown that in both human and mouse CD8^+^ T cells, ACC impedes lipid utilization by CD8^+^ T cells in the TME, ultimately leading to intracellular lipid droplet accumulation and fat degeneration. Inhibition of ACC to eliminate fatty acid synthesis and storage in CD8^+^ T cells enhances the utilization of free fatty acids by mitochondria to sustain bioenergetics, resulting in prolonged anti-tumor immunity. This is reflected by a significant enrichment of gene sets associated with T cell memory, a reduction in exhaustion markers, an increase in multifunctional cytokine production, and better maintenance of mitochondrial structural dynamics 67. Interestingly, another report indicates that long-chain fatty acids, such as linoleic acid (LAs), activate genes related to lipid droplets in CD8^+^ T cells. This stimulation promotes the formation of lipid droplets and lipid storage, enhances both fatty acid synthesis and oxidation, and boosts the metabolic adaptability of memory CD8^+^ T cells. Furthermore, triglycerides stored in lipid droplets can serve as a source of fatty acids for FAO in memory T cells 75. Together, these findings suggest that moderate lipid droplet storage may support FAO, whereas excessive lipid synthesis and lipid droplet accumulation may suppress FAO, underscoring the complex role of lipid metabolism in regulating CD8⁺ T cell function and memory. Furthermore, peroxisome proliferator-activated receptors (PPARs) belong to the lipogenic gene family. In a murine melanoma model, CD8^+^ TILs enhanced PPARα signaling and fatty acids catabolism under conditions of hypoglycemia and hypoxia. This metabolic switch partially preserved the effector functions of CD8^+^ TILs and improved their ability to slow tumor progression 76. In another study, it was reported that METRNL exerts its effects on mitochondrial respiration through the PPARδ-E2F pathway in CD8^+^ T cells 61.

#### Fatty acid transport

Fatty acid transport proteins involved in lipid metabolism also influence FAO in CD8^+^ T cells. Fatty acid transport protein (FATP) is responsible for transporting intracellular fatty acids to organelles such as mitochondria or the endoplasmic reticulum for fatty acid metabolism 77. In an *in vitro* multiple myeloma model, bone marrow CD8^+^ T cells exhibited impaired mitochondrial function and immune activity due to lipid uptake mediated by FATP1 expression 78. Additionally, CD36, a scavenger receptor that facilitates the transport of fatty acids and oxidized lipids, has been reported to show increased expression in tumor-infiltrating CD8⁺ T cells, which is associated with cancer progression and poor survival in both human and murine models 79. Beyond its role in transporting oxidized low-density lipoprotein (ox-LDL), cholesterol in the TME can upregulate CD36 expression in CD8^+^ T cells, leading to excessive fatty acid uptake. This process triggers lipid peroxidation damage and ferroptosis, resulting in the loss of cytotoxic function and promoting tumor growth 80. Furthermore, fatty acid transporters involved in lipid metabolism also affect the FAO of CD8^+^ T cells. Fatty acid binding protein 5 (FABP5) provides fuel for mitochondrial respiration by coordinating lipid uptake to maintain the bioenergy requirements of CD8^+^ T cells 76, 77. Recent studies have shown that in patients with ovarian cancer, the expression of transgelin 2 (TAGLN2) in cytoskeleton tissue is inhibited, leading to the inability of FABP5 to be effectively transported to the cell surface, which affects the lipid uptake and mitochondrial respiration of CD8^+^ T cells. In the mouse model of ovarian cancer, overexpression of TAGLN2 in CAR-T cells can restore the function of FABP5, enhance lipid uptake and mitochondrial respiration, and thus improve the anti-tumor activity of CAR-T cells 24.

#### Ferroptosis

Ferroptosis is an iron-dependent form of cell death driven by lipid peroxidation. Mechanisms such as intracellular iron accumulation, elevated levels of ROS and lipid peroxides, and inhibition of the glutathione-glutathione peroxidase 4 (GSH-GPX4) pathway are interconnected and collectively influence the fate of CD8⁺ T cells. An increase in intracellular free iron (Fe²⁺) is a hallmark of ferroptosis. Iron is taken up by cells via the transferrin/transferrin receptor system. Excess Fe²⁺ acts as a “catalyst” in the Fenton reaction, converting otherwise harmless intracellular hydrogen peroxide or lipid peroxides into highly reactive hydroxyl radicals or lipid radicals. These radicals “steal” hydrogen atoms from polyunsaturated fatty acids, initiating and amplifying a chain reaction of lipid peroxidation, thereby acting as a “fuel” for ferroptosis 81. For instance, as mentioned above, lipid accumulation in the TME induces high expression of CD36 in CD8⁺ T cells, promoting their lipid peroxidation and ferroptosis. Similarly, PD-1 signaling in CD8⁺ Tex cells suppresses the expression of phospholipid phosphatase 1 (PLPP1) via the Akt-GATA1 pathway, and the accumulation of unsaturated fatty acids (such as arachidonic acid and octadecenoic acid) in the TME further drives ferroptosis in PLPP1^lo^ CD8⁺ T cells. Overexpression of PLPP1 may represent a novel strategy to enhance the anti-tumor function of CD8⁺ T cells 82.

However, CD8⁺ T cells possess a robust antioxidant system to counteract lipid peroxidation, the core of which is GPX4. The activity of GPX4 relies on GSH, and it is the only enzyme capable of directly reducing phospholipid hydroperoxides within cell membranes. Studies have shown that cystine is the rate-limiting substrate for GSH synthesis. CD8⁺ T cells are at a disadvantage in competing for cystine within the TME; cystine deficiency leads to reduced GSH synthesis, inhibition of the GSH-GPX4 pathway, and subsequently promotes exhaustion and ferroptosis in CD8⁺ T cells, impairing their anti-tumor function. Supplementing cystine to increase GPX4 levels may be a potential therapeutic strategy to enhance the anti-tumor efficacy of CD8⁺ T cells 83.

#### Cholesterol metabolism

Mitochondrially generated acetyl-CoA is transported to the cytoplasm and endoplasmic reticulum, where it undergoes enzymatic reactions to synthesize cholesterol. Similar to lipid droplets, changes in cholesterol levels can have heterogeneous effects on T cell function. On one hand, the cholesterol content in tumor-infiltrating CD8^+^ T cells is positively correlated with the expression of exhaustion markers such as PD-1, 2B4, TIM-3, and LAG-3, and this accumulation of cholesterol ultimately leads to T cell exhaustion. Lowering cellular cholesterol levels has been shown to restore the effector function of CD8^+^ TILs 84. Consistent with this, cholesterol has been reported to inhibit TCR signaling at the early activation stage of CD8^+^ T cells by directly limiting the movement of the TCR-CD3 core complex 85. Interestingly, statins, widely used to lower cholesterol synthesis, only reduce cholesterol levels in CD8^+^ T cells before activation; however, post-activation, simvastatin treatment increases cholesterol levels in CD8^+^ T cells 86. Furthermore, statins also inhibit CD8^+^ T cell activation and proliferation 87, indicating that statins are not potential anti-tumor therapeutic agents targeting T cell cholesterol metabolism. However, fibroblast growth factor 1 intracellular binding protein (Fibp) knockout CD8^+^ T cells maintain their tumor-killing efficacy even after cholesterol treatment, suggesting that the inhibitory effect of cholesterol on CD8^+^ T cell function depends on FIBP 86. On the other hand, a recent study showed that in CD8^+^ TILs, oxysterols mediate the reciprocal alteration of the liver-X receptor (LXR) and sterol regulatory element binding protein 2 (SREBP2) pathways, leading to cholesterol deficiency. This cholesterol deficiency stress inhibits CD8^+^ T cell proliferation and induces autophagy-mediated apoptosis, driving CD8^+^ T cell exhaustion and dysfunction 88. In line with this, the inhibition of Acetyl-CoA Acetyltransferase 1 (ACAT1), a key enzyme involved in cholesterol esterification, increased cholesterol content in the membrane of CD8^+^ T cells, thereby enhancing TCR clustering, signaling, and immunological synapse formation, ultimately improving their anti-tumor capacity 89. In addition to the cholesterol effects within CD8^+^ T cells, the cholesterol content in the TME also affects the function of TILs. Cholesterol in the TME exists in lipoprotein forms, primarily low-density lipoprotein (LDL) and high-density lipoprotein (HDL). It has been reported that the CD36 receptor on CD8^+^ T cells promotes the uptake of ox-LDL into the cells, inducing lipid peroxidation and activation of downstream p38 kinase, which results in CD8^+^ T cell dysfunction in TILs 79. In contrast, LDL and HDL promote T cell proliferation; in particular, LDL can mediate transcriptional reprogramming via the low-density lipoprotein receptor-related protein11-mitogen-activated protein kinase13-TCF (LRP11-MAPK13-TCF) axis to facilitate the activation and proliferation of CD8⁺ TIL cells 90. These findings highlight the dual role of cholesterol in regulating T cell function within the TME and the potential for targeting lipid metabolism pathways to modulate T cell responses in cancer therapy.

To sum up, the impact of fatty acids and their derived lipids on CD8^+^ TILs is environment-dependent and influenced by various factors, such as their intracellular concentrations, location, chemical form, and the complexity of their metabolism and processing. This process involves the differentiation, functional regulation, and survival of CD8^+^ T cells in different physiological and pathological environments. By modulating lipid metabolism, it is possible to influence the biological behavior of T cells, providing new approaches and strategies for the treatment of immune-related diseases.

### Amino acid metabolism

Amino acids are considered one of the basic building blocks of life. In addition to being the fundamental components of proteins, amino acid metabolism also participates in various other cellular processes to regulate cell function. In CD8^+^ T cells, amino acids are essential for activation and differentiation 91-93. Interestingly, restricting the intake of these amino acids in the TME enhances CD8^+^ T cell immune responses 94, 95. It is widely believed that the excessive uptake of amino acids by tumor cells creates an amino acid-deprived environment for immune cells, particularly CD8^+^ T cells, hindering the normal function of these critical immune cells. For example, tumor cells compete with CD8^+^ T cells for methionine uptake by overexpressing SLC43A2. which leads to methionine deficiency in CD8^+^ T cells and impairs the formation of histone methylation, especially the loss of dimethylation at lysine 79 of histone H3 (H3K79me2), resulting in decreased expression of STAT5 and impaired immune function of CD8^+^ T cells 96.

#### Glutamine

Glutamine is essential for CD8^+^ T cell activation and function, including indirect involvement in the TCA cycle, providing a nitrogen source for the synthesis of amino acids and nucleotides in cells, and maintaining mTOR1 activity along with other amino acids 97. For example, activated CD8^+^ T cells regulate glutamine-dependent mitochondrial metabolism via AMP-activated protein kinase (AMPK) to maintain their energy supply 98. Interestingly, despite glutamine's role in regulating CD8^+^ T cell differentiation, activation, and function, blocking glutamine metabolism in the TME does not weaken T cell function as expected but instead enhances CD8^+^ T cell anti-tumor activity 94. Earlier, it was believed that excessive glutamine uptake by tumor cells limits glutamine availability for infiltrating immune cells, driving metabolic adaptation and preventing effective activation of anti-tumor CD8⁺ T cells 99. Recent studies, however, have proposed an alternative mechanism. In mammalian cells, glutamine utilization can be mediated by two enzymes: nicotinamide adenine dinucleotide phosphate (NADP⁺)-dependent IDH1 in the cytosol and IDH2 in the mitochondria. Proliferating effector CD8⁺ T cells utilize the mitochondrial enzyme IDH2 to reductively carboxylate glutamine, thereby epigenetically locking CD8⁺ T lymphocytes into a terminal effector differentiation program. [U-13C]-glutamine kinetic labeling *in vitro* shows that effector CD8^+^ T cells consume more glutamine than memory CD8^+^ T cells and are more proliferative. *In vitro* experiments indicate that the deletion of the gene encoding IDH2, which inhibits glutamine production, does not impair CD8^+^ T cell proliferation or effector function, but promotes the differentiation of memory CD8^+^ T cells. This finding could be used to optimize the therapeutic efficacy of CAR-T cell therapy 100.

It is worth noting that recent studies have shown that ammonia produced from glutamine contributes to the death of effector CD8^+^ T cells. The specific mechanism is that rapidly proliferating CD8^+^ T cells utilize glutamine metabolism to release ammonia in the mitochondria, which is then transported and stored in lysosomes. Excess ammonia accumulation increases the pH of the lysosome, leading to the termination of ammonia storage in the lysosome and the backflow of ammonia into the mitochondria, causing mitochondrial damage and cell death. This process is characterized by lysosomal alkalinization, mitochondrial swelling, and impaired autophagic flux. Inhibition of glutamine metabolism or blocking lysosomal alkalinization can prevent ammonia-induced CD8^+^ T cell death and improve CD8^+^ T cell-based cancer immunotherapy. Overall, it is currently widely accepted that glutamine in the TME acts as an immunosuppressive factor for CD8^+^ T cells 101.

#### Tryptophan

Unlike glutamine, there is significant disagreement regarding the role of tryptophan metabolism in T cells within the TME. Tryptophan metabolism is predominantly carried out via three major pathways: the kynurenine pathway, the serotonin pathway, and the indole pathway 102. Over 95% of free tryptophan is metabolized by indoleamine 2,3-dioxygenase (IDO) into kynurenine, which is further broken down into other metabolites through various kynureninases, such as melatonin, quinolinic acid, kynurenic acid, tryptamine, vitamin B3, NAD^+^, and NADP^+^ 103. Some studies suggest that tryptophan metabolism directly promotes T cell activation and proliferation. The specific mechanism is that tryptophan is metabolized into indole-3-propionic acid (IPA) under the influence of gut microbiota. IPA can increase the acetylation of H3K27 in the super-enhancer region of the TCF7 in CD8^+^ T cells, regulating the stemness program of CD8^+^ T cells and promoting the generation of CD8^+^ Tpex cells, thereby enhancing the efficacy of anti-PD-1 immunotherapy 19. However, other studies suggest that in the TME, overexpression of IDO leads to the accumulation of tryptophan metabolites, which indirectly suppress Teff cell function by activating Tregs and impairing dendritic cell function, thus promoting tumor progression and immune escape 104. Additionally, IFN-γ secreted by CD8^+^ T cells stimulates the upregulation of IDO1 expression in tumor cells, resulting in high levels of kynurenine. Kynurenine is then transferred to adjacent CD8^+^ T cells via the transporters solute carrier family 7 member 8 (SLC7A8) and proton-coupled amino acid transporter 4 (PAT4), inducing and activating the AhR, which in turn upregulates PD-1 expression 105. The serotonin pathway is another important route of tryptophan metabolism and has been implicated in pro-inflammatory responses 106, 107. CD8⁺ T cells synthesize serotonin by expressing tryptophan hydroxylase 1 (TPH1) and release it into the TME. Serotonin accumulates in the TME and enhances the activity of CD8⁺ T cells by activating serotonin receptors (5-HTR). The serotonin transporter (SERT) reuptakes extracellular serotonin into cells, thereby reducing serotonin levels in the TME. SERT inhibitors can block SERT activity, leading to increased serotonin levels in the TME, further activating 5-HTR, enhancing the effector function and proliferative capacity of CD8⁺ T cells, and ultimately boosting the anti-tumor immune response 108. Another study found that intracellularly accumulated serotonin (5-HT) in CD8⁺ T cells, catalyzed by transglutaminase 2, induces serotoninylation of GAPDH. This modification promotes the translocation of GAPDH from the nucleus to the cytoplasm, thereby enhancing glycolytic metabolism and further promoting the activation of CD8⁺ T cells and their anti-tumor immune activity 109.

#### Methionine

Methionine metabolism involves three main pathways: the methionine cycle, the transsulfuration pathway, and the methionine salvage cycle 110. In the methionine cycle, the intermediate metabolite S-adenosylmethionine (SAM) is a key methyl donor for nucleotide methylation and epigenetic modifications. Several studies have shown that disruption of the methionine cycle, due to reduced SAM levels, impairs CD8^+^ T cell activation. Additionally, other intermediate metabolites in the methionine cycle, such as S-adenosylhomocysteine (SAH) and homocysteine (Hcy), also affect T cell activation 111. In CD8^+^ T cells cultured in the supernatant of tumor cells, both SAM and SAH levels are significantly decreased, while supplementing methionine restores the levels of these metabolites, thereby improving their function 96.

Spermidine (SPD), a derivative of the transsulfuration pathway, not only promotes the differentiation of T cells into memory subsets and enhances their cytotoxicity but also restores the function of CD8⁺ T cells in aged mice by promoting fatty acid oxidation. The transsulfuration pathway generates sulfur-containing metabolites, including GSH and hydrogen sulfide (H2S) 112-114. GSH, a byproduct of the methionine salvage pathway, is a common cellular antioxidant that effectively clears excess ROS. By overexpressing relevant enzymes to enhance intracellular GSH synthesis and replenishment, ROS levels in CD8⁺ Tex cells can be reduced, representing a promising strategy to rejuvenate their function and enhance T cell-based immunotherapy 60, 115, 116. Additionally, H2S enhances CD8^+^ T cell survival and activity by increasing the sulfur hydration of GAPDH and promoting glycolysis 117.

Methionine is an essential amino acid that must be obtained from the diet 96, 118, 119. In addition to the aforementioned requirement of methionine for T cell activation and differentiation, tumor cells also require large amounts of methionine to maintain protein synthesis, redox homeostasis, regulate methylation reactions, sulfur amino acid metabolism, and signaling pathways, among other functions 17, 118. The impact of methionine restriction (MR) on tumor growth and treatment response depends not only on the tumor cells' own dependency on methionine but also on the relative reliance of cancer cells and T cells on methionine within the TME. Reports indicate that in CD8^+^ T cells, tumor-induced disruption of methionine metabolism depletes intracellular methionine and reduces the availability of the methyl donor SAM. The resulting loss of the histone mark H3K79me2 suppresses STAT5 expression, leading to impaired T-cell immune function 96. Currently, several anti-tumor drugs targeting methionine metabolism pathways are in clinical stages due to the high activity of methionine metabolism in tumor cells. However, pre-feeding with an MR diet has been shown to increase the growth of subcutaneously transplanted melanoma tumors in wild-type C57BL/6J mice 117, but not in CD8a knockout C57BL/6J mice. Additionally, short-term methionine deprivation can induce ferroptosis in tumor cells by upregulating the transcription of cation transport regulator homolog 1 (CHAC1), making tumor cells more susceptible to CD8^+^ T cell-mediated cytotoxicity and enhancing the synergistic effect with ICB therapy. In contrast, prolonged MR can inhibit CHAC1 expression and prevent ferroptosis in tumor cells 83. Based on these findings, it has been speculated that in healthy individuals or early-stage cancer patients, T cells are more dependent on methionine, and methionine restriction may suppress T cell activation, leading to an inability to control tumor growth or respond to immunotherapy 111. However, in advanced cancer patients, tumor cells may have a greater dependence on methionine, and methionine restriction could act synergistically with non-immune-mediated therapies to inhibit tumor progression 120.

Therefore, factors such as the timing, duration, and extent of methionine restriction, the stage and type of cancer, and the immune status of the patient could all influence the tumor's response to methionine restriction-based treatment. These results further highlight the need to consider potential physiological, pathological, and environmental factors when targeting methionine metabolism pathways in therapy.

#### Other amino acids

In addition to glutamine, tryptophan, and methionine, other amino acids such as cysteine, arginine, serine and leucine are also considered to play important roles in CD8^+^ T cell expansion, differentiation, and function.

Within the TME, most tumor cells or certain immunosuppressive cells overexpress solute carrier (SLC) transporters, which play a crucial role in amino acid transport. As a result, the competitive uptake of amino acids by tumor and immunosuppressive cells creates an amino acid-deprived environment for CD8^+^ T cells, hindering their normal function. For example, tumor cells preferentially uptake cystine by overexpressing Slc7a11, leading to insufficient cystine uptake by CD8^+^ T cells, which in turn impairs glutathione synthesis, disrupts cystine-glutamate exchange, and triggers T cell exhaustion and ferroptosis. Overexpressing the catalytic subunit of glutamate-cystine ligase (Gclc) in CD8^+^ T cells can promote glutathione synthesis, thereby enhancing T cell anti-tumor function. Therefore, Gclc could serve as a potential therapeutic target to boost T cell anti-tumor activity 83. Similarly, in breast cancer, tumor-associated macrophages (TAMs) uptake large amounts of arginine via assisted transport (SLC7A1/SLC7A2) or reverse transport (SLC7A6/SLC7A7), synthesize proline, and secrete ornithine, which can impair the metabolism and anti-tumor activity of CD8^+^ T cells within TAMs. In addition, extracellular serine promotes Teff cell proliferation through serine, glycine, and one carbon metabolic networks 121, 122. Supplementing with serine can rescue the decreased proliferation ability of CD8^+^ T cells induced by lactate 41. Furthermore, leucine, isoleucine, and valine, essential amino acids that cannot be synthesized *de novo* by the human body, are collectively known as branched-chain amino acids (BCAAs). In mice deficient in the type 2 serine/threonine protein phosphatase (PP2Cm), the accumulation of BCAAs in CD8^+^ T cells enhances effector functions and anti-tumor responses by reprogramming glucose metabolism. High levels of BCAAs also promote human CD8^+^ T cell function and may improve clinical responses to anti-PD-1 therapy 123. Besides, it has been reported that knocking in the key enzyme proline dehydrogenase 2 (PRODH2) involved in the proline metabolism pathway in CAR-T cells leads to a transition towards OXPHOS, manifested by an increase in mitochondrial count, oxygen consumption rate, spare respiratory capacity, and a decrease in extracellular acidification rate 124.

Generally speaking, regulating amino acid metabolism reprogramming—such as controlling the concentrations of free amino acids, their membrane-bound transporters, key metabolic enzymes, and sensors—can influence the phenotype and function of CD8^+^ T cells. Therefore, enhancing anti-cancer immune responses can be achieved by supplementing specific essential amino acids or targeting transporters, metabolic enzymes, or their sensors, thus paving the way for the development of new adjuvant immunotherapy strategies.

#### Crosstalk among metabolic signaling pathways

The complex cross-regulation between glucose, lipid, and amino acid metabolic pathways is a hallmark of cellular metabolism. This intricate network enables cells to maintain efficient energy homeostasis and adapt to nutrient fluctuations, ensuring that metabolic intermediates from one pathway can serve as substrates, signals, or regulators for another. Key molecules such as acetyl-CoA, α-KG, and NADPH act as universal connectors within this network. Acetyl-CoA is a core hub in CD8^+^ T cell metabolism, sitting at the convergence point of glucose, lipid, and amino acid metabolism. Enzymes including acetyl-CoA synthetase (ACSS2), ATP-citrate lyase (ACLY), and acetyl-CoA carboxylase (ACC) coordinate carbon flux, ultimately determining CD8^+^ T cell differentiation fate. ACSS2 utilizes acetate to promote acetate-dependent histone acetylation, fostering the formation of self-renewing CD8^+^ Tpex cells. ACLY metabolizes citrate to drive citrate-dependent histone acetylation, promoting the differentiation of CD8^+^ Tex cells 125. ACC channels acetyl-CoA into lipogenesis, leading to lipid droplets accumulation which impairs the effector function of CD8^+^ Teff cells. Conversely, ACC inhibition promotes mitochondrial FAO, supporting the development of CD8^+^ Tm cells 72.

α-KG resides at the intersection of metabolic reprogramming, epigenetic modification, and cell signaling in CD8^+^ T cells. While primarily derived from glutaminolysis, its homeostasis is supported by energy and TCA cycle intermediates from glucose and lipid metabolism 126. Glutaminase (GLS1) and IDH2 coordinately regulate α-KG flux to determine cell fate. Research shows that xylulose-5-phosphate from the PPP activates the carbohydrate response element binding protein (ChREBP) pathway, upregulating glutaminase synthesis and driving α-KG production. As a cofactor for Tet methylcytosine dioxygenase (TET) enzymes, α-KG facilitates TET3 binding to the Tcf7 promoter, inducing DNA demethylation and increasing H3K4me3 enrichment. This sustains Tcf7 expression, expands the Tpex pool, and suppresses Tex differentiation 127. Similarly, inhibiting IDH2 restores the normal function of α-KG, relieving inhibition on histone demethylases like KDM5. This increases H3K4me3 and H3K27ac levels, inducing the formation of Tm cells with enhanced anti-tumor capacity 128.

As a central coenzyme and reductant, NADPH is involved in antioxidant defense, biosynthetic processes, and redox signaling. Its levels directly impact the anti-tumor efficacy of CD8^+^ T cells. The PPP is a primary source of NADPH. In Tm cells, glycogenolysis-derived glucose-1-phosphate (G1P) promotes the liquid-liquid phase separation of G6PD and other PPP enzymes, enhancing NADPH generation. Nanoparticle-targeted delivery of G1P has been shown to restore NADPH production via the PPP, reduce ROS levels, and thereby improve the efficacy of CAR-T therapy and cancer vaccines 129. NADPH also links lipid metabolism to ferroptosis. PD-1 signaling in Tex cells suppresses PLPP1 expression via the Akt-GATA1 axis. Subsequently, unsaturated fatty acids (e.g., arachidonic acid, octadecenoic acid) accumulating in the TME drive ferroptosis in PLPP1^lo^ CD8^+^ T cells 82. Supporting this, gene set enrichment analysis (GSEA) of RNA-seq data from CD8^+^ T cells in early-stage lung cancer patients revealed that PLPP1^hi^ populations are enriched for pathways involving NADPH regeneration and the negative regulation of ROS metabolism 82.

In summary, post-translational modifications—such as acetylation, methylation, lactylation, and succinylation—that are derived from metabolites represent a key mechanism integrating metabolic intermediates with immune signaling pathways. This critical link between metabolism and immunoregulation offers promising therapeutic avenues for cancer. Future cancer treatments will likely require precisely matching metabolic interventions with immunotherapies based on the specific metabolic profile of a patient's tumor.

## Discussion

In summary, metabolic reprogramming of CD8⁺ T cells in the TME leads to alterations in the epigenetic landscape and signaling pathways, thereby affecting CD8⁺ T cell differentiation, proliferation, survival, and antitumor immunity. Existing research consensus indicates that there is no single, absolutely central metabolic pathway. The core metabolic pathways relied upon by different CD8⁺ T cell subsets are highly heterogeneous and vary with the spatiotemporal dynamics of the TME and therapeutic interventions. Specifically: glycolysis is the core pathway for Teff cells; Tpex cells depend on the TCA cycle; Tm cells predominantly utilize FAO; while in Tex cells, lipid peroxidation drives ferroptosis, and OXPHOS is closely associated with the maintenance of their exhausted state. The characteristics of different mitochondrial metabolic pathways in CD8⁺ T cells within the TME are outlined in Table 3.

Due to space limitations, this review focuses only on three major metabolic pathways in CD8⁺ T cells: glucose metabolism, lipid metabolism, and amino acid metabolism. It does not delve deeply into other metabolic types, such as nucleotide metabolism, one-carbon metabolism, and mannose metabolism. In fact, these pathways also play significant and heterogeneous roles in the activation, differentiation, and function of CD8⁺ T cells. For example, asparagine influences nucleotide synthesis by regulating nuclear factor erythroid 2-related factor 2 (NRF2) expression, ultimately affecting CD8⁺ T cell proliferation 64. The folate metabolism enzyme inhibitor pemetrexed, when combined with ICB for NSCLC treatment, can upregulate PD-L1 expression and restore Tex-cell activity via the Thymidylate Synthase (TYMS)-ROS-NF-κB regulatory axis 130. Sarcosine dehydrogenase inhibits NF-κB signaling and CD8⁺ T cell function by promoting sarcosine degradation in one-carbon metabolism and increasing SAM consumption, thereby suppressing H3K79me2 modification of NF-κB-activated genes 131. D-mannose treatment restores mannose metabolism in CD8⁺ T cells and increases O-GlcNAc transferase-mediated O-GlcNAcylation of β-catenin, maintaining Tcf7 expression and epigenetic stemness to promote stem-like programs in T cells 132. These non-canonical metabolic pathways in CD8⁺ T cells demonstrate considerable research value and application potential in cancer therapy.

**Figure 3:**
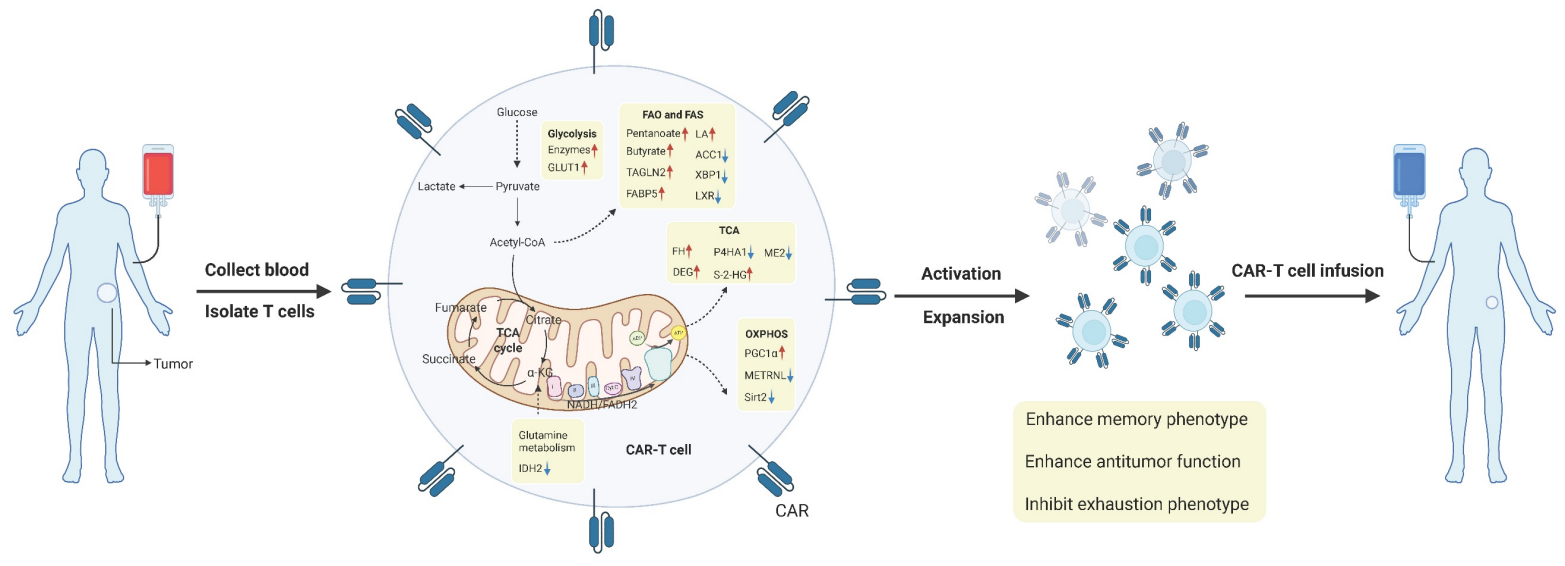
CAR-T therapy enhances anti-tumor effect by regulating the metabolism of CD8+ T cells. 1. Collect peripheral blood and isolate and purify T cells. 2. Using genetic engineering technology to transform T cells into CAR-T cells. 3. Expand and activate CAR-T cells* in vitro*. 4. The expanded CAR-T cells were reinfused to the patient via vein to start tumor cell immunotherapy. ACC1: acetyl-CoA carboxylase 1; CAR: chimeric antigen receptor; DEG: diethyl 2-hydroxyglutarate; FABP5: fatty acid binding protein 5; FAO: fatty acid oxidation; FAS: fatty acid synthesis; FH: fumarate hydratase; GLUT1: glucose transporter 1; IDH2: isocitrate dehydrogenase 2; LA: linoleic acid; LXR: liver-X receptor; ME2: malic enzyme 2; METRNL: meteorin-like protein; OXPHOS: oxidative phosphorylation; PGC-1α: peroxisome proliferator-activated receptor-gamma coactivator-1α; S-2-HG: S-2-hydroxyglutaric acid; Sirt2: sirtuin 2; TAGLN2: transgelin 2; TCA: tricarboxylic acid; XBP1: X-box binding protein 1.

Targeting metabolic checkpoints in CD8⁺ T cells represents a highly promising approach to improving T cell-based anti-tumor immunotherapy. According to recent research findings, certain metabolites and enzymes, such as IDH2, PAHA1, fumarate, VLCFAs have been shown to significantly suppress the anti-tumor capacity of CD8⁺ T cells. However, the heterogeneous effects of the same metabolites within the TME on CD8⁺ T cell function and differentiation pose a major challenge to tumor immunotherapy. Notable examples include lactate, succinate, ROS, tryptophan, methionine, among others. To address this issue, we hypothesize that, firstly, the concentration of metabolites is a key determining factor—excessive accumulation of metabolites can disrupt CD8⁺ T cell homeostasis and thereby induce exhaustion. Secondly, CD8⁺ T cells themselves exist in different differentiation and metabolic states, each possessing distinct metabolic programs and signaling pathways. Lastly, the multifunctionality of metabolites themselves plays a role; some key metabolites can serve as energy substrates to support T cell survival while also acting as signaling molecules to influence cell differentiation through epigenetic pathways. Overall, CD8⁺ T cells are a highly heterogeneous population in terms of functional states, with non-uniform metabolic statuses, signaling pathways, and epigenetic backgrounds. Therefore, the same metabolite can produce markedly different, or even opposite, effects depending on its concentration, timing of action, and the subtype and metabolic state of the CD8⁺ T cell. At present, we have not yet fully elucidated their subtypes or the associations between subtypes and metabolic characteristics. For example, PD-1^+^ CD8⁺ T cell activation inhibits glucose uptake and glycolysis while promoting fatty acid oxidation 133-136. Thus, a deeper understanding of the metabolic reprogramming mechanisms in CD8⁺ T cells and other cells within the TME will provide new insights and targets for metabolic intervention. Improving the survival rate, functional persistence, and ability of CD8⁺ T cells to overcome metabolic stress in the TME constitutes the second major challenge for CD8⁺ T cell immunotherapy. Current studies indicate that some metabolic intervention strategies show significant advantages in enhancing CD8⁺ T cell-mediated anti-tumor immunity. For instance, modifying or genetically engineering CAR-T or TCR-T cells to reshape T cell metabolism, inhibiting terminal differentiation of CD8⁺ T cells, promoting the formation of long-lived memory CD8⁺ T cells, enhancing T cell proliferation, and increasing sensitivity to tumor antigens can improve therapeutic outcomes 22. Notably, the basic principle of CAR-T therapy involves genetically engineering a patient's own T cells to equip them with a “guidance head” (CAR) that specifically recognizes tumor antigens, enabling precise tumor cell killing. The basic process of CAR-T cell synthesis is illustrated in Figure 3. Currently, CAR-T cells have been approved for patients with B-cell malignancies or relapsed and/or refractory multiple myeloma. In recent years, the clinical efficacy of CAR-T therapy in solid tumors has also improved significantly, though it has not yet been applied in clinical treatment for solid tumors 137-141. One of the principal limitations lies in the method of CAR-T cell construction: the use of viral vectors can lead to permanent CAR expression, which may result in potential serious side effects. To address this limitation, studies have shown that transient CAR expression in T cells via mRNA has emerged as a promising new strategy 142. Another key challenge involves the efficient delivery of CAR-T cells into solid tumors. Recent research indicates that hydrogels can serve as a delivery platform for CAR-T cells, significantly enhancing their tumor infiltration and persistence in solid tumor therapy 143. It is important to note that most current research on novel metabolic checkpoints remains at the *in vitro* stage. *In vitro* culture conditions cannot accurately replicate the metabolic changes occurring in the physiological TME, including factors such as nutrient availability, cell-cell interactions, and cytokine environments. Therefore, metabolically targeted pathways identified *in vitro* require careful validation *in vivo*, and more advanced research techniques and deeper methodological approaches are needed to bridge these gaps, such as the broader application of organoid models in cancer research. This represents the third challenge we face. Additionally, since tumor cells, immunosuppressive cells, and CD8⁺ T cells competitively uptake certain metabolites, the anti-tumor effect of targeting these metabolites depends on the relative dependency of tumor cells, immunosuppressive cells, and T cells within the TME on these metabolites. Another major issue is that current metabolically targeted therapies struggle to achieve stable and long-term anti-tumor effects. The discovery of more effective and broadly applicable metabolic checkpoints is essential for advancing cancer immunotherapy.

In summary, metabolic checkpoint intervention strategies show significant potential in enhancing CD8⁺ T cell function, extending their survival, and overcoming the immunosuppressive microenvironment. However, several challenges remain: including the concentration- and spatiotemporal-dependency of metabolite effects, the heterogeneity of CD8⁺ T cell states, the gap between *in vitro* models and the *in vivo* microenvironment, and the limited efficacy and difficulty in sustaining long-term responses of current therapies in solid tumors. Currently, the understanding of immune reprogramming mechanisms in different cells under normal physiological conditions and within the TME at various stages of progression remains superficial and fragmented. In-depth study of metabolic reprogramming and the establishment of a more comprehensive, three-dimensional, and dynamic metabolic reprogramming network may provide innovative and effective therapeutic strategies for cancer patients. In the future, through a profound understanding of the spatial heterogeneity and temporal dynamics of the TME metabolism, we are expected to move beyond “one-size-fits-all” therapies and innovatively design multidimensional precision treatment plans tailored to individual patients, bringing revolutionary breakthroughs to the next generation of immune-metabolic combination therapies.

### Future challenges and outlook

In the current wave of precision medicine, the advent of disruptive technologies such as single-cell multi-omics and spatial metabolomics has propelled our understanding of the TME into a new “high-resolution” era 144. This has catalyzed a highly promising innovative direction in cancer therapy: integrating artificial intelligence (AI) technology to precisely select the most compatible metabolic checkpoint inhibitors and immunotherapeutic agents based on the specific metabolic profile of a patient's TME, and enabling accurate drug delivery to the tumor site 145.

Specifically, we first need to construct a panoramic metabolic atlas of CD8⁺ T cells across different tumor types through integrated analysis of single-cell transcriptomics, epigenomics, proteomics, and metabolomics. This includes comparing “hot” tumors like kidney cancer, rich in T-cell infiltration, with “cold” tumors like pancreatic cancer, which lack such infiltration. Within the same tumor type, spatial metabolomics can be employed to map a “metabolic landscape” visualizing the actual distribution of key metabolites (e.g., lactate, ROS) across distinct functional regions such as the tumor core, invasive front, and tertiary lymphoid structures. This allows for the precise correlation of CD8⁺ T cell subsets and their metabolic features with their specific anatomical niches within the tumor tissue, thereby identifying “metabolism-specific checkpoints”. It is crucial to utilize novel immunotherapies, such as adoptive transfer of TILs, CAR-T or TCR-T cell therapies 26, 95, 146, and inhibitors targeting immune checkpoints 147 and metabolic checkpoints 30. Among these, CAR-T cell therapy represents a significant breakthrough in recent years. Subsequently, Lipid nanoparticle (LNP) delivery technology can be leveraged to precisely transport these immunotherapeutic/metabolic checkpoint-related drugs or CAR-T cells to the target lesions, enhancing their efficacy and durability. Currently, LNP drug delivery technology is steadily advancing. LNP-mediated mRNA-based cancer vaccines can effectively activate the immune system and amplify anti-tumor immune responses. LNP delivery systems can also transport mRNA-encoded antibodies directly to tumor sites, enabling precise targeting. Furthermore, mRNA-encoded cytokines delivered via LNPs can modulate immune responses, further augmenting anti-tumor effects. Additionally, using the CRISPR-Cas9 system, LNP delivery can introduce gene-editing tools into T cells, offering therapy at the genetic level. Research indicates that mRNA-LNP technology demonstrates favorable safety and efficacy profiles in clinical trials targeting advanced cholangiocarcinoma, colorectal cancer, and triple-negative breast cancer 148. Moreover, integrating chronobiology research to administer therapies at specific time points—synergizing with endogenous metabolic fluctuations—can optimize the timing, duration, and intensity of immunotherapeutic interventions. This approach enhances efficacy while mitigating toxic side effects. For instance, administering immune checkpoint inhibitors within the transient fluctuation window of specific metabolites (e.g., ATP, potassium ions) induced by chemotherapy or radiotherapy can maximally activate CD8⁺ T cells 149.

Notably, recent studies have shown that the microbiota can regulate antitumor immunity 150, 151. In particular, the gut microbiota has attracted broader attention 152, 153, and how it shapes the immune and metabolic functions of CD8⁺ T cells have become an emerging focus. For instance, Cao *et al.* reported that an intact gut microbiota can cooperate with ICIs to increase CD8⁺ T-cell abundance while decreasing their dependence on glycolytic metabolism. Through this metabolic reprogramming, CD8⁺ Tex cells are shifted toward memory and effector phenotypes, thereby enhancing antitumor immunity 154. Lam *et al.* demonstrated that the gut microbiota can produce stimulator of interferon genes (STING), which remodels the anti-tumor TME and may render it more conducive to CD8⁺ T-cell function 155. Sivan *et al.* further reported that the effect of commensal Bifidobacterium in promoting antitumor immunity and improving anti-PD-L1 efficacy is mediated by enhanced dendritic cell function, which in turn strengthens CD8⁺ T-cell priming and supports their accumulation in the TME 156. With respect to the mechanisms by which the gut microbiota regulates CD8⁺ T cells, recent work has highlighted gut microbe-driven epigenetic regulation mediated by microbiota-derived metabolites 157. For example, butyrate increases H3K27ac at the promoter regions of Pdcd1 and Cd28 in human CD8⁺ T cells, thereby promoting PD-1/CD28 expression and enhancing the efficacy of anti-PD-1 therapy 158. Indole derivatives provide another metabolite-based example. Jia *et al.* revealed that Lactobacillus johnsonii collaborates with Clostridium sporogenes to generate IPA. IPA influences the stemness program of CD8⁺ T cells and promotes the formation of CD8⁺ Tpex cells by increasing H3K27ac at the Tcf7 super-enhancer region 19. Beyond epigenetic regulation, microbiota-derived metabolites can also directly shape the antitumor capacity of CD8⁺ T cells. A compelling example is succinate produced by the gut bacterium Fusobacterium nucleatum, which impairs CD8⁺ T-cell immunity. By suppressing the cyclic GMP-AMP synthase (cGAS)-interferon-β signaling pathway, succinate reduces the efficacy of anti-PD-1 monoclonal antibody therapy and contributes to immunotherapy resistance in colorectal cancer 159. At the level of therapeutic application, available evidence indicates that interventions such as fecal microbial transplant, prebiotics, probiotics, and related strategies that remodel the gut microbial landscape in cancer patients may facilitate the expansion of beneficial commensals and their metabolites within the intestinal lumen, thereby augmenting antitumor immune responses, including those mediated by CD8⁺ T cells 160-164. Future studies are needed to further determine whether changes in the immune and metabolic functions of CD8⁺ T cells represent a central component of microbiota-related therapeutic efficacy.

In summary, the comprehensive integration of single-cell multi-omics and spatial metabolomics for high-resolution profiling, along with engineered cellular therapies such as CAR-T, LNP-enabled spatiotemporal drug delivery, chronotherapy informed by biological rhythms, and systemic modulation of the gut microbiota, will allow us to more fully map and target therapeutically actionable metabolic nodes within CD8⁺ T cells in the TME. Together, these approaches pave the way toward a multidimensional precision medicine framework tailored to individual patient profiles. By uniting precise spatiotemporal intervention with holistic systemic regulation, this strategy promises to catalyze a paradigm shift in immune-metabolic combination therapy, offering both an innovative conceptual framework and actionable tools for earlier diagnosis, more precise treatment, and improved prognosis in oncology.

## Figures and Tables

**Table 1:** Differences and interconnections between metabolic checkpoints and immune checkpoints

Features	Metabolic checkpoints	Immune checkpoints
Level of action	At the level of cellular metabolism.	At the level of intracellular signal transduction.
Main function	Regulate the energy sources and biosynthetic pathways of CD8⁺ T cells, thereby governing their functional differentiation and cell-fate decisions.	Regulate the activation state of CD8⁺ T cells by delivering inhibitory or stimulatory signals, thereby preventing excessive immune responses and immune evasion.
Mechanism of action	By competitively consuming key nutrients (e.g., glucose, amino acids) and accumulating immunosuppressive metabolic by-products (e.g., excessive ROS, lactate), metabolic checkpoints disrupt the metabolic adaptability of CD8⁺ T cells and indirectly suppress their antitumor activity.	Inhibitory receptors expressed on CD8⁺ T cells (e.g., PD-1, CTLA-4) bind to their ligands and directly transmit inhibitory signals, thereby directly suppressing the antitumor activity of CD8⁺ T cells.
Fate of CD8⁺ T cells	Distinct metabolic pathways regulate the differentiation of naïve CD8⁺ T cells into effector, memory, precursor exhausted, and terminally exhausted subsets, among others.	Engagement of different inhibitory receptors with their ligands activates signaling pathways that directly drive CD8⁺ T cells into a state of terminal exhaustion.
Key targets	LDHA; acetyl-CoA; ROS; glutamine.	PD-1; CTLA-4; LAG-3; TIM-3; TIGIT.
Representative therapies	Metabolic checkpoint inhibitors: MCT1 inhibitors (e.g., AZD3965); antioxidants (e.g., N-acetylcysteine).	Immune checkpoint inhibitors: anti-PD-1/PD-L1 antibodies (e.g., pembrolizumab); anti-CTLA-4 antibodies (e.g., ipilimumab).
Interrelationship	Mutual reinforcement and synergy: (1) Metabolism as the basis of signaling: metabolic reprogramming of T cells is fundamental for their activation and effector functions; metabolic inhibition can attenuate the efficacy of immune checkpoint inhibitors. (2) Signaling regulates metabolism: activation of immune checkpoints (e.g., PD-1) directly suppresses glycolysis and other metabolic processes in CD8⁺ T cells. (3) Convergence on T-cell exhaustion: chronic metabolic stress together with sustained inhibitory signaling jointly drive CD8⁺ T cells toward terminal exhaustion.	

CTLA-4: cytotoxic T-lymphocyte associated protein 4; LDHA: lactate dehydrogenase A; LAG-3: lymphocyte activation gene-3 protein; MCT1: monocarboxylate transporter 1; PD-1: programmed death 1; PD-L1: programmed death-1 ligand 1; ROS: reactive oxygen species; TIM-3: T-cell immunoglobulin and mucin-domain containing-3; TIGIT: T cell immunoreceptor with Ig and ITIM domains.

**Table 2:** Clinical trials of strategies targeting metabolic dysfunction in intratumoral CD8⁺ T cells

Metabolic pathway	Metabolic checkpoint	Related agent	Mechanism of action	Tumor type	NCT number and clinical phase	Ref
Glucose metabolism	mTOR	IL-7Rα CAR-T cells (mTOR agonists)	IL-7 and IL-15 activate the mTOR signaling pathway, enhance glycolysis in CD8⁺ T cells, and promote differentiation into Tscm cells.	Solid tumors; glioma	NCT06612645 (Phase I); NCT06221553 (Phase I)	165-167
		IL-15- and IL-21-armored CAR-T cells (mTOR agonists)	IL-21 and IL-15 act synergistically to simultaneously increase IFN-γ production and significantly enhance the proliferation of both memory and naïve phenotype CD8^+^ T cells.	Solid tumors; liver cancer; acute lymphoblastic leukemia	NCT06198296 (Phase I); NCT04715191 (Phase I); NCT07148050 (Phase I); NCT04377932 (Phase I); NCT06783816 (unclear)	168-170
	MCT1	AZD3965 (MCT1 inhibitor)	LC activates PKCθ signaling, promoting relocalization of MCT1 to mitochondria and thereby facilitating mitochondrial uptake of lactate.	Colorectal cancer; breast cancer; lung cancer	NCT01791595 (Phase I)	171
	IDH2	Enasidenib (IDH2 inhibitor)	The IDH2 inhibitor enhances glucose utilization and cytosolic acetyl-CoA levels, thereby promoting histone acetylation in CD8⁺ Tm cells.	Chondrosarcoma; sinonasal carcinoma; neuroblastoma	NCT06176989 (Phase II)	47
		HMPL-306 (IDH2 inhibitor)	Same as above.	Glioma	NCT07025018 (Phase I)	172
OXPHOS	ROS	Acetyl cysteine (antioxidant)	Inhibits excessive ROS production and thereby prevents CD8⁺ T cells from progressing toward a terminally exhausted state.	Solid tumors; myeloproliferative neoplasms	NCT02569957 (Phase II); NCT05123365 (Phase I/II); NCT05081479 (Phase I)	173
		GSH	GSH is a ubiquitous intracellular antioxidant that efficiently scavenges excessive ROS in CD8⁺ T cells.	NSCLC	NCT06896422 (Phase I)	111
	AMPK	Metformin (AMPK agonist)	AMPK is a key activator of mitophagy; its activation clears dysfunctional, ROS-overproducing “old” mitochondria and promotes the generation of new mitochondria, thereby improving the overall quality of the mitochondrial pool in CD8⁺ T cells.	Diffuse large B-cell lymphoma	NCT02531308 (Phase II)	56
Lipid metabolism	PPAR	Pioglitazone (PPARγ agonist)	PPAR directly upregulates genes involved in fatty acid uptake and storage in CD8⁺ T cells, thereby promoting the differentiation of Tm cells.	Melanoma; NSCLC; Hepatocellular Carcinoma	NCT04114136 (Phase II)	76
		CS7017 (PPARγ agonist)	Same as above.	Anaplastic Thyroid Cancer	NCT00603941 (Phase I/II)	61
Amino acid metabolism	Glutaminase	CB-839 (glutaminase inhibitor) plus nivolumab	Blockade of glutamine metabolism within the TME promotes differentiation of CD8⁺ T cells into Tscm cells rather than Tex cells.	ccRCC; melanoma; NSCLC; solid tumors	NCT02771626 (Phase I/II); NCT03872427 (Phase II); NCT02071862 (Phase I); NCT03965845 (Phase I/II); NCT03875313 (Phase I/II)	94
	IDO	NLG802 (IDO inhibitor)	IFN-γ secreted by CD8⁺ T cells upregulates IDO1 expression in tumor cells, leading to high levels of kynurenine, which are transported into neighboring CD8⁺ T cells via SLC7A8 and PAT4, inducing and activating the AhR and thereby upregulating PD-1.	Solid tumors	NCT03164603 (Phase I)	105
		IDO peptide vaccination (IDO inhibitor)	Same as above.	NSCLC; lung cancer	NCT01219348 (Phase I); NCT03047928 (Phase I/II); NCT05077709 (Phase II)	
		GDC-0919 (IDO inhibitor)	Same as above.	Solid tumors	NCT02048709 (Phase I)	
		DN1406131 (IDO inhibitor)	Same as above.	Solid tumors	NCT03641794 (Phase I)	
		SHR9146 (IDO inhibitor)	Same as above.	Solid tumors	NCT03491631 (Phase I); NCT03208959 (Phase I)	
		IO102-IO103 (IDO inhibitor)	Same as above.	Melanoma; lung cancer; NSCLC	NCT05155254 (Phase II); NCT05077709 (Phase II)	
		Indoximod (IDO pathway inhibitor)	Same as above.	Glioma; metastatic melanoma	NCT02502708 (Phase I); NCT02073123 (Phase I/II); NCT02077881 (Phase I/II)	
		Epacadostat (IDO inhibitor)	Same as above.	Solid tumors	NCT01685255 (Phase II)	
	SAM	SAM	Early supplementation of Met during CD8⁺ T-cell activation restores the SAM cycle and methylation of arginine 350 (R350) on the Ca^2+^-activated potassium transporter KCa3.1, reduces Ca²⁺ influx and NFAT1 nuclear localization, and thereby restrains T-cell exhaustion.	Hepatocellular carcinoma	NCT05701553 (unclear); NCT02586285 (Phase I); NCT03178929 (unclear); NCT00513461 (Phase II)	111
		S095035 (MAT2A inhibitor)	Inhibits tumor-cell growth and remodels the metabolic state of the TME, thereby alleviating TME-mediated suppression of CD8⁺ T cells.	Solid tumors	NCT06188702 (Phase I/II)	174
		IDE397 (MAT2A inhibitor)	Same as above.	Solid tumors	NCT04794699 (Phase I)	

AhR: aryl hydrocarbon receptor; AMPK: AMP-activated protein kinase; CAR: chimeric antigen receptor; ccRCC: clear-cell renal cell carcinoma; GSH: glutathione; IDH: isocitrate dehydrogenase; IDO: indoleamine 2,3-dioxygenase; IFN-γ: interferon-gamma; IL-7: interleukin-7; IL-7Rα: interleukin-7 receptor-alpha; IL-15: interleukin-15; IL-21: interleukin-21; LC: lithium carbonate; MAT2A: methionine adenosyltransferase 2A; MCT1: monocarboxylate transporter 1; mTOR: mechanistic target of rapamycin; Met: methionine; NFAT1: nuclear factor of activated T cells 1; NSCLC: non-small cell lung cancer; PAT4: proton-coupled amino acid transporter 4; PKCθ: protein kinase C θ; PPAR: peroxisome proliferator-activated receptor; ROS: reactive oxygen species; SAM: S-adenosyl-methionine; SLC7A8: solute carrier family 7 member 8; Tex: exhausted T; Tm, memory T; TME: tumor microenvironment; Tscm: stem cell memory T.

**Table 3:** Characteristics of distinct mitochondrial metabolic pathways in CD8⁺ T cells within the TME

Metabolic pathway	Main features	Impact on CD8⁺ T cells	Key metabolites/enzymes	Cellular localization
TCA cycle	(1) Occupies a central position in cellular energy metabolism; (2) acetyl-CoA lies at the core of energy metabolism and connects the three major metabolic pathways; (3) TCA cycle intermediates also exert signaling functions.	(1) Core metabolic pathway in Tpex cells; (2) an important metabolic pathway for Tm cells.	Key metabolites: acetyl-CoA, citrate, succinate, fumarate, α-KG; key enzymes: IDH2, SDH, P4HA1, ME2, α-KGDD.	Mitochondrial matrix
OXPHOS	(1) Utilizes reducing equivalents (NADH, FADH2) generated by the TCA cycle to drive the electron transport chain and produce ATP; (2) major source of ROS within CD8⁺ T cells.	(1) Core metabolic mechanism underlying exhaustion in Ttex cells; (2) important metabolic pathway in Tpex cells.	Key metabolites: ROS, NAD⁺/NADH, PGC-1α; key enzymes: SIRT1, SIRT2.	Inner mitochondrial membrane
FAO	(1) Lipid catabolism generates acetyl-CoA, which enters the TCA cycle; (2) multiple metabolites have signaling functions and contribute to membrane biogenesis; (3) exerts markedly different effects on the functions of distinct CD8⁺ T cell subsets.	(1) An important energy source for resting CD8⁺ T cells; (2) core metabolic pathway for CD8⁺ Tm cells and a key energy source supporting their long-term survival and maintenance of memory function; (3) important metabolic pathway in Tpex cells; (4) critical metabolic pathway involved in ferroptosis of Ttex cells.	Key metabolites: SCFAs, LCFAs, VLCFAs, TAGs, lipid droplets, cholesterol; key enzymes: FABP5, TAGLN2, VLCAD.	Mitochondrial matrix
Glutamine metabolism	(1) Provides anaplerotic substrates for the TCA cycle; (2) supplies precursors for the synthesis of key biomacromolecules such as proteins and nucleotides; (3) exerts distinct effects on the functions of different CD8⁺ T cell subsets	(1) Provides energy and biosynthetic precursors for activated CD8⁺ T cells; (2) accumulation of ammonia, a by-product of glutamine metabolism, leads to mitochondrial damage and cell death in CD8⁺ T cells.	Key metabolites: glutamine, glutamate, ammonia; key enzymes: AMPK, glutaminase.	Mitochondrial matrix

ATP: adenosine triphosphate; α-KG: α-ketoglutarate; α-KGDD: α-KG-dependent dioxygenases; AMPK: AMP-activated protein kinase; FABP5: fatty acid binding protein 5; FADH2: flavin adenine dinucleotide dihydrogen; FAO: fatty acid oxidation; IDH2: isocitrate dehydrogenase 2; LCFAs: long-chain fatty acids; ME2: malic enzyme 2; NAD^+^: nicotinamide adenine dinucleotide; NADH: nicotinamide adenine dinucleotide hydride; OXPHOS: Oxidative phosphorylation; P4HA1: prolyl 4-hydroxylase 1; PGC-1α: peroxisome proliferator-activated receptor-gamma coactivator-1α; ROS: reactive oxygen species; SCFAs: short-chain fatty acids; SDH: succinate dehydrogenase; SIRT: sirtuin; TAGLN2: transgelin 2; TAGs: triacylglycerols; TCA: tricarboxylic acid; Tm: memory T; Tpex: precursor exhausted T; Ttex: terminally exhausted T; VLCAD: very long-chain acyl-CoA dehydrogenase; VLCFAs: very long-chain fatty acids.
